# ACOD1 regulates microglial arginine metabolism and inflammatory responses

**DOI:** 10.3389/fimmu.2026.1731962

**Published:** 2026-03-16

**Authors:** Eleftheria Karadima, Canelif Yilmaz, Anupam Sinha, Georgia Fodelianaki, Sofia Dimothyra, Nikolaos Nirakis, Sofia Traikov, Nicola Zamboni, Ben Wielockx, Panayotis Verginis, Mirko Peitzsch, Triantafyllos Chavakis, Vasileia Ismini Alexaki

**Affiliations:** 1 Institute for Clinical Chemistry and Laboratory Medicine, Faculty of Medicine and University Hospital Carl Gustav Carus, Technische Universität Dresden, Dresden, Germany; 2 Institute of Molecular Systems Biology, Eidgenössische Technische Hochschule (ETH) Zurich, Zurich, Switzerland; 3 Laboratory of Immune Regulation and Tolerance, Division of Basic Sciences, Medical School, University of Crete, Heraklion, Greece

**Keywords:** ACLY, ACOD1, argininosuccinate, microglia, polyamines

## Abstract

Itaconate is produced by inflammatory macrophages and promotes negative feedback on inflammation. It is synthesized by aconitate decarboxylase 1 (ACOD1) from cis-aconitate, a metabolite of the tricarboxylic acid cycle. Here, we focused on the role of ACOD1 in the immunometabolic reprograming of inflammatory microglia. Similar to macrophages, ACOD1 deficient microglia displayed a stronger inflammatory response to lipopolysaccharide (LPS) compared to their wild type counterparts. The proinflammatory effects of ACOD1 deficiency were associated with enhanced ATP citrate lyase (ACLY) activity and elevated acetyl-CoA amounts, and reprogramed arginine metabolism entailing enhanced argininosuccinate synthesis at the expense of polyamine biosynthesis. These effects of ACOD1 deficiency on arginine metabolism were reversed by ACLY inhibition. These findings provide new insights in the immunometabolic role of ACOD1.

## 1 Introduction

Macrophage immune responses are orchestrated by cell metabolic reprograming (1). Itaconate, a byproduct of the tricarboxylic acid cycle (TCA), is produced in inflammatory macrophages and negatively feedbacks on inflammation (2). It is synthesized through decarboxylation of cis-aconitate by aconitate decarboxylase 1 [ACOD1, encoded by Immune responsive gene 1 (*Irg1* or *Acod1*)], the expression of which is induced in macrophages by inflammatory stimuli, such as lipopolysaccharide (LPS) (3). Itaconate reprograms the TCA cycle by inhibiting succinate dehydrogenase (SDH), leading to accumulation of succinate (4, 5). Several molecular mechanisms mediate the anti-inflammatory effects of itaconate including 1) alkylation of KEAP1 and downstream activation of Nuclear factor erythroid 2-related factor 2 (NRF2), 2) alkylation-mediated inhibition of stimulator of interferon genes (STING), 3) Activating Transcription Factor 3 (ATF3)-mediated inhibition of IκBζ and 4) inhibition of TET-family DNA dioxygenases and thereby downregulation of NF-κB and STAT target genes (6–9). Consequently, itaconate reduces the expression of pro-inflammatory cytokines, such as IL-1β, and IL-6, and downregulates production of reactive oxygen species (ROS) (5). The anti-inflammatory function of the ACOD1-itaconate axis was shown in infection, sepsis, myocardial disease, atherosclerosis, autoimmune disease and gout (7, 8, 10–15). However, intriguingly, itaconate was also shown to promote inflammatory responses in tissue-resident alveolar macrophages (16).

Microglia are the resident macrophage-like cells of the central nervous system (CNS) (17). They assist in proper synaptic remodeling (18) and maintain brain homeostasis via removal of damaged or dead cells and debris (19). In neurodegenerative diseases, microglia lose their homeostatic function and acquire an inflammatory phenotype (20). Sustained low-grade microglia inflammation is a common feature of many neurological diseases, such as Alzheimer’s disease and multiple sclerosis (MS) (21). Cell metabolism orchestrates microglia function but the involved cell metabolic circuits are still inadequately understood (22). Here, we investigated the role of ACOD1 in the immunometabolic reprograming of microglia upon LPS stimulation. In accordance with previous studies, we show that ACOD1 expression is induced in inflammatory microglia and that ACOD1 deficiency exacerbates microglia inflammation (23–26). To stimulate microglia inflammation we used systemic LPS administration (27). Furthermore, we uncover a novel mechanism of action of ACOD1, which involves regulation of ATP citrate lyase (ACLY) and downstream tuning of arginine metabolism. Particularly, we show that ACOD1 deficiency increases ACLY activity and acetyl-CoA levels, and reprograms arginine metabolism towards the pro-inflammatory argininosuccinate synthesis at the expense of the anti-inflammatory polyamine metabolism in an ACLY-dependent manner.

## 2 Materials and methods

### 2.1 Mice and *in vivo* experiments

*Acod1^-/-^* mice were purchased from The Jackson Laboratory (JAX #029340) and bred with wild type (wt) C57BL/6J mice. In all experiments littermate *Acod1^-/-^* and wt mice were used. Wt C57BL/6J mice were obtained from Charles River Laboratories. Eight to twelve-week old male mice were intraperitoneally (i.p.) injected with 3 mg/kg LPS (LPS-EB Ultrapure; InVivoGen, tlrl-3pelps) or PBS and after 4, 16 or 24 hours they were sacrificed by cervical dislocation. In other experiments, wt C57BL/6J mice were i.p. injected with 50 mg/kg argininosuccinate, or PBS and after 3 h 1 mg/kg LPS was i.p. injected. Four h later mice were killed by cervical dislocation. All animal experiments were in compliance with the local ethical guidelines and approved by Landesdirektion Sachsen, Germany.

### 2.2 Microglia isolation, culture and treatments

Primary microglia were isolated as previously described (27, 28). Briefly, mouse brains of 8–9 week-old littermate wt and *Acod1*^-/-^ mice were digested with an enzymatic solution containing 0.5 mM EDTA, 5 mM L-cysteine (Sigma-Aldrich), 0.1 mg/ml papain (Sigma-Aldrich), and 2.4 mg/ml dispase II (Sigma Aldrich) diluted in DMEM (Thermo Fisher Scientific). The enzymatic reaction was stopped by adding 20% FBS in PBS. After centrifugation at 1,200 rpm for 7 min at 4 °C, cells were re-suspended in 0.5 mg/ml DNase I (Thermo Fisher Scientific) in PBS and incubated for 5 min in room temperature (RT). Cells were gently dissociated and passed through a 100 μm cell strainer. Isolated cells were cultured in DMEM/F12 (Thermo Scientific) with Glutamax, 10% FBS, 1% penicillin/streptomycin (P/S) and 10 ng/ml granulocyte and macrophage colony stimulating factor (GM-CSF) (Peprotech) in poly-L-Lysine-coated flasks and maintained in culture at 37 °C and 5% CO_2_. Cells were treated with LPS (100 ng/ml, tlrl-peklps, InvivoGen), Interferon γ (IFN-γ, 20 ng/ml, Thermo Fisher Scientific), BMS303141 (20 μM, Sigma-Aldrich), spermidine (10 μM, Sigma-Aldrich) or respective vehicle controls.

### 2.3 Isolation of glial populations and neurons

Mouse brains were dissected and the Neural Tissue Dissociation Kit (Miltenyi Biotec) was used for obtaining single-cell suspensions according to manufacturer’s instructions. Sequential isolation of microglia, oligodendrocytes and astrocytes from the same samples was achieved by positive selection after serial incubation with anti-CD11b Microglia Microbeads (Miltenyi Biotec), anti-O4 Microbeads (Miltenyi Biotec) and anti-astrocyte cell surface antigen-2 (ACSA-2) Microbeads kit (Miltenyi Biotec) with sequential passage through LS columns. Neurons were isolated by negative selection for CD11b, O4 and ACSA-2.

### 2.4 FACS sorting of brain microglia

Brains were smashed in isolation buffer (0.5% BSA PBS) on ice and cell suspensions were centrifuged at 300 x g for 10 min at 4 °C. Myelin was removed with Myelin Removal Beads II (Miltenyl Biotec) and LS columns (Miltenyl Biotec) per manufacturer’s instructions. The cells were incubated with anti-CD45-PerCPCy5.5 (1:100, 103132, Biolegend), anti-CD11b-FITC (1:100, 101206, Biolegend) or anti-Ly6G-APC (1:100, 560599, BD Biosciences) in 5% FBS PBS for 30 min at 4 °C in the dark. Microglia were identified as CD45^interm^CD11b^+^Ly6G^-^. Microglia were sorted with a BD FACSAria II (BD Biosciences) and the FACSDiva software (BD Biosciences). Sorted cells were collected in 10% FBS in PBS, centrifuged at 1,400 rpm for 10 min at 4 °C and the cell pellet was stored at -80 °C until further analysis.

### 2.5 BV2 cell culture and treatments

BV2 cells were obtained from Interlab Cell Line Collection (ICLC, Genova, Italy) and maintained in RPMI-1640 medium supplemented with 10% FBS and 1% P/S at 37 °C and 5% CO_2_. BV2 cells were treated for 4 h with following TLR ligands: PAM3CSK4 (TLR1/TLR2 ligand, 1 μg/ml), heat-killed preparation of Listeria monocytogenes (HKLM, TLR2 ligand, 10^8^ cells/ml), polyinosinic-polycytidylic acid (poly(I:C), TLR3 ligand, 1 μg/ml), flagellin from Salmonella Typhimurium (FLA-ST, TLR5 ligand, 1 μg/ml), FSL-1 (TLR2/6 ligand, 100 ng/ml), imiquimod (TLR7 ligand, 1 μg/ml), ssRNA40/Lyovec (TLR8 ligand, 1 μg/ml) and Class B CpG oligonucleotide (ODN2006, TLR9 ligand, 5 μM), all from the Human TLR1–9 Agonist kit (InvivoGen, tlrl-kit1hw). Also, BV2 cells were treated for 4 h with M-CSF, GM-CSF, IL-1β, IL-6, tumor necrosis factor (TNF), IL-4, IL-10, Transforming Growth Factor β (TGF-β) or IFN-γ (all at 20 ng/ml from Peprotech).

### 2.6 siRNA transfections

Primary microglia cells were transfected with small interfering RNAs (siRNAs) and Lipofectamine™ RNAiMAX Transfection Reagent (Invitrogen) using the forward transfection protocol according to the manufacturer’s protocol. Cells were incubated with 30 nM siRNAs for 24 h (si*Ass1* and siControl) or 48 h (si*Odc* and siControl). All siRNAs were purchased from Dharmacon-Horizon Discovery.

### 2.7 RNA-seq

Bulk RNA-seq was performed and analyzed as previously described (29, 30). For transcriptome mapping, strand-specific paired-end sequencing libraries from total RNA were constructed using TruSeq stranded Total RNA kit (Illumina Inc). Sequencing was performed on an Illumina HiSeq3000 (1x75 basepairs). Low quality nucleotides were removed with the Illumina fastq filter and reads were further subjected to adaptor trimming using cutadapt (31). Alignment of the reads to the mouse genome was done using STAR Aligner (32) using the parameters: “–runMode alignReads –outSAMstrandField intronMotif –outSAMtype BAM SortedByCoordinate --readFilesCommand zcat”. Mouse Genome version GRCm38 (release M12 GENCODE) was used for the alignment. The parameters: ‘htseq-count -f bam -s reverse -m union -a 20’, HTSeq-0.6.1p1 (33) were used to count the reads that map to the genes in the aligned sample files. The GTF file (gencode.vM12.annotation.gtf) used for read quantification was downloaded from Gencode (https://www.gencodegenes.org/mouse/release_M12.html). Gene centric differential expression analysis was performed using DESeq2_1.8.1 (34). The raw read counts for the genes across the samples were normalized using ‘rlog’ command of DESeq2 and subsequently these values were used to render a PCA plot using ggplot2_1.0.1 (35).

Pathway and functional analyses were performed using GSEA (35). GSEA is a stand-alone software with a graphical user interface (GUI). To run GSEA, a ranked list of all the genes from DESeq2 based calculations was created using the -log10 of the p-value. This ranked list was then queried against GO and Reactome based repositories.

### 2.8 RNA isolation and quantitative RT-PCR

Total RNA was extracted from cells or tissues using the Nucleospin RNA isolation kit (Macherey-Nagel), according to manufacturer’s instructions. cDNA was synthesized using the iScript cDNA synthesis kit (Bio-Rad). qPCR was performed using the SsoFast Eva Green Supermix (Bio-Rad), a CFX384 real-time System C1000 Thermal Cycler (Bio-Rad), and the Bio-Rad CFX Manager 3.1 software. The relative amount of mRNA was calculated with the ΔΔCt method, using *18s* as a housekeeping gene. The primer sequences are listed in **Table 1**.

**Table 1:** Primer sequences.

Gene name	Primer	Sequence (5' to 3')
*18s*	forward	GTTCCGACCATAAACGATGCC
*18s*	reverse	TGGTGGTGCCCTTCCGTCAAT
*Acod1*	forward	CTCCCACCGACATATGCTGC
*Acod1*	reverse	GCTTCCG TAGAGCTGTGA
*Il-1b*	forward	TGGGATGATGATGATAACCTGC
*Il-1b*	reverse	TCGTTGCTTGGTTCTCCTTGTA
*Il-6*	forward	CCTTCCTACCCCAATTTCCAAT
*Il-6*	reverse	AACGCACTAGGTTTGCCGAGTA
*Ass1*	forward	CTGCTATTCACTGGCACCCC
*Ass1*	reverse	GATCATTTCGGCCCTTGAACC
*Sall1*	forward	GCTTGCACTATCTGTGGAAGAGC
*Sall1*	reverse	CTGGGAACTTGACAGGATTGCC
*Tmem119*	forward	GTGTCTAACAGGCCCCAGAA
*Tmem119*	reverse	AGCCACGTGGTATCAAGGAG
*Cx3cr1*	forward	AGGACACAGCCAGACAAG
*Cx3cr1*	reverse	TCAGGGGAGAAAGCAAG
*Trem2*	forward	GTACTGGTGGAGGTGCTGGA
*Trem2*	reverse	GGAGGTGCTGTGTTCCACTT
*Mertk*	forward	CGGTAATAATCACCACTGTAAATCTTTCT
*Mertk*	reverse	TTGCGGGATGACATGACTGT
*Nos2*	forward	ACCTTGTTCAGCTACGCCTT
*Nos2*	reverse	CATTCCCAAATGTGCTTGTC
*Odc1*	forward	CGCAGTCAAGTGTAACGATAGC
*Odc1*	reverse	GAGACTTGTTTACAAGGATTTGCAT
*Acly*	forward	AGGAAGTGCCACCTCCAACAGT
*Acly*	reverse	CGCTCATCACAGATGCTGGTCA
*Slc25a1*	forward	GGAGGCACACAAATACCGGA
*Slc25a1*	reverse	GGTGCCCTTGTAGAATGCCT

### 2.9 Cut&Tag

Cut&Tag (Active Motif) was performed according to manufacturer’s instructions. Cells were centrifuged at 600 x g for 3 min at RT, followed by wash with 1X Wash Buffer and centrifugation at 600 x g for 3 min. Samples were kept on ice after preparation. Then, 20 μl of Concanavalin-A magnetic beads were added to each sample and samples were incubated for 10 min at RT on an end-over-end rotator. Samples were placed on a magnetic stand to clear for 2 min. Next, they were incubated with 50 μl of ice-cold antibody buffer containing 1 μg/ml primary antibody anti-histone H3K9ac (Active motif, 39918) or rabbit (DA1E) mAb IgG XP^®^ isotype control (Cell signaling, 3900s) overnight at 4 °C with orbital mixing. Afterwards, the samples were placed on a magnetic stand to clear for 2 min and they were incubated in 100 μl of Dig-Wash buffer containing 1:100 guinea pig anti-rabbit secondary antibody at RT for 60 min with orbital mixing, followed by 3 washes with 1 ml Dig-Wash Buffer. Then, in order for the assembled pA-Tn5 Transposomes to form, the samples were incubated with 100 μl of Dig-300 Buffer, containing 1:100 Cut&Tag-IT™ Assembled pA-Tn5 Transposomes at RT for 60 min with orbital mixing, followed by 3 washes with 1 ml Dig-300 Buffer. Afterwards, the samples were incubated with 125 μl of Tagmentation Buffer for 60 min at 37 °C. In order to stop the tagmentation and solubilize the DNA fragments, 4.2 µl 0.5 M EDTA, 1.25 µl 10% SDS and 1.1 µl Proteinase K (10 mg/mL) were added to each sample, followed by 60 min incubation at 55 °C. Next, the samples were placed in a magnetic stand to clear for 2 min and 625 μl of DNA purification Binding Buffer were added to each sample. The samples were transferred to a DNA Purification Column and centrifuged at 17,000 x g for 1 min. The flow-through was discarded and 750 µl of DNA Purification Wash Buffer were added to the column. The samples were centrifuged at 17,000 x g for 1 min and the flow-through was discarded. The empty tubes were centrifuged again at 17,000 x g for 2 min to remove any remaining DNA Purification Wash Buffer. Sixty μl of DNA Purification Elution Buffer were added to the center of the column matrix, samples were incubated at RT for 1 min and centrifuged at 17,000 x g for 1 min to collect the DNA. Quantitative PCR was performed using 1 μl of eluted DNA for each reaction. The relative amount of Cut&Tag enriched fragment was calculated with the ΔΔCt method. The primer sequences are listed in **Table 2**.

**Table 2:** Primer sequences.

Gene name	Primer	Sequence (5' to 3')
*Ass1* (sorted microglia)	forward	GAGAGGGTGCATCTTTCCCA
*Ass1* (sorted microglia)	reverse	GAGCCACTTTGAGGCCATTG
*Ass1* (primary microglia)	forward	CACCTCTGTGAACCTCAACCT
*Ass1* (primary microglia)	reverse	CCATTTTAACGTCCTGGCCT

### 2.10 Non-targeted metabolomics

Cells were washed with 75 mM ammonium carbonate at pH 7.4 and cell pellets were collected and frozen in liquid nitrogen. Intracellular metabolites were extracted twice with 70% ethanol at 75 °C for 3 min, dried in a speedvac and resuspended in H_2_O. Extracts were analyzed by flow injection – time of flight mass spectrometry on an Agilent 6550 QTOF instrument, as described previously (36). Ion annotation was based on matching their measured masses to that of the compounds listed in the KEGG mmu database.

### 2.11 Targeted metabolomics

TCA metabolites were measured as previously described (37). Briefly, metabolites were extracted from samples with methanol, dried, resuspended in mobile phase and cleared with a 0.2 µm centrifugal filter. To improve separation, the elution gradient was changed as follows: 99% A (0.2% formic acid in water), 1% B (0.2% formic acid in acetonitrile) for 2.00 min, 100% B at 2.50 to 2.65 min, 1% B at 3.40 min and equilibration with 1% B until 5.00 min. Multiple reaction monitoring with negative electrospray ionization was used for quantification. Itaconate was measured using multi-reaction monitoring (MRM)-derived ion transition of 128.9→85.1. For quantification of itaconate ratios of analyte peak areas to respective peak areas of the stable isotope labeled internal standard (itaconic acid-^13^C5; Bio-Connect B.V., The Netherlands; MRM transition 133.9→89.1) obtained in samples were compared to those of calibrators.

Arginine metabolites were measured as previously described (38). Sample preparation was performed by addition of 10 µl internal standard working solution followed by 200 µL H_2_O:acetonitrile 50:50 (v/v) extraction buffer and subsequent grinding for 30 sec. After homogenization, samples were vortex-mixed for one minute and centrifuged at 3,000g for 10 min at 4 °C. Clear supernatants were transferred directly onto a 96-well- polytetrafluoroethylene (PTFE)-filterplate (Merck-Millipore) and filtered by assistance of positive pressure. Subsequently, filtered extracts were dried in a vacuum-assisted centrifuge, thereafter reconstituted in 200 µl initial mobile phase and analyzed by LC-MS/MS. LC-MS/MS measurements were performed on a QTRAP^®^ 6500+ triple quadrupole mass spectrometer from Sciex coupled to a Waters Acquity ultra-performance liquid chromatography system. Chromatographic separation was achieved by using a XBridge BEH Amide XP Column (2.1 x 100 mm, 2.5 µm; Waters) at 40 °C using a gradient of mobile phases A (20mM ammonium formate/5% methanol at pH 3) and B (ACN/methanol/mobile phase A, 90%/5%/5%). Five µL of reconstituted calibrators, QC samples and test samples, kept at 4 °C in the autosampler, were injected into the LC-MS/MS system at a flow rate of 0.4 mL/min with 15% mobile 29 phase A. At 0.37 min, mobile phase A started to linearly increase up to 30% at 4.1 min and further to 50% at 5 min. At 5.8 min, mobile Phase A increased up to 85% and after a hold until 6.8 min, the gradient returned back to initial conditions at 7.8 min, followed by another 1.7 min for column re-equilibration.

### 2.12 Acetyl-CoA measurement

Acetyl-CoA was measured as previously described (29). Cultured cells were washed with cold PBS, scraped on ice in cold PBS, centrifuged for 2 min at 1,500 rpm at 4 °C, washed once with 0.1 M ammonium bicarbonate, and centrifuged again for 2 min at 1,500 rpm at 4 °C. Cell pellets and tissues were snap-frozen and stored at -80 °C until further analysis. Frozen samples were dissolved in 150 μl of 30% methanol in acetonitrile containing 100 nM AMP – isotope-labeled (Adenosine-¹³C_10_,¹^5^N_5_-5′-monophosphate) as an internal standard. LC–MS/MS analysis was performed using a high-performance liquid chromatography (HPLC) system (Agilent 1200) coupled online to a G2-S QTof mass spectrometer (Waters). For normal-phase chromatography, Bridge Amide 3.5 μm (2.1 × 100 mm) columns (Waters) were used. The mobile phase consisted of eluent A (95% acetonitrile, 0.1 mM ammonium acetate, and 0.01% NH_4_OH) and eluent B (40% acetonitrile, 0.1 mM ammonium acetate, and 0.01% NH_4_OH), applied with the following gradient program: 0% to 100% eluent B within 18 min, 100% eluent B from 18 to 21 min and 0% eluent B from 21 to 26 min. The flow rate was set to 0.3 ml/min. The spray voltage was set to 3.0 kV, and the source temperature was maintained at 120 °C. Nitrogen was used as both the cone gas (50 l/h) and desolvation gas (800 l/h), while argon was used as the collision gas. The MSE mode was applied in negative ionization polarity. Mass chromatograms and spectral data were acquired and processed using MassLynx software (Waters).

For the measurement of ACLY activity, primary microglia cells were incubated or not with 100 μM ^13^C-Citrate (LGC, TRC-C521004) for 0.5, 2 and 5 h in DMEM/F12 without FBS. ^13^C-acetyl-CoA measurement was performed as described above for acetyl-CoA. Adenosine-¹³C_10_,¹^5^N_5_-5′-monophosphate was used as an internal standard. Results were normalized to the protein content, which was determined using the BCA method (Thermo Fisher Scientific).

### 2.13 Western blotting

Protein extracts were prepared in ice-cold RIPA lysis buffer system supplemented with protease and phosphatase inhibitors (SCBT) or lysis buffer supplemented with PhosSTOP (Roche) and cOmplete^™^, Mini, EDTA-free Protease Inhibitor Cocktail (Roche) Protein concentration was determined with the BCA assay (Thermo Fisher Scientific). Protein lysates were mixed with reducing Laemmli SDS sample buffer (Thermo Fisher Scientific), denatured at 95 °C for 5 min and loaded on a polyacrylamide gel and separated with SDS-PAGE. Afterward, proteins were transferred onto nitrocellulose membranes and blocking was performed with 5% BSA TBS-T buffer for 1 h at RT followed by overnight incubation with the primary antibody. Primary antibodies used were following: anti-ACOD1 (Abcam, ab222411), anti-IL-1β (Cell Signaling Technology, #12507), anti-phospho-ACLY (Cell Signaling Technology, #4331S), anti-β-actin (Cell Signaling Technology, #4970), anti-Vinculin (Cell Signaling Technology, #4650) and anti-Tubulin (Sigma-Aldrich, T5186) all diluted at 1:1,000 in 5% BSA TBS-T. Next, goat anti-rabbit IgG horseradish peroxidase-conjugated antibody (1:3,000, R&D Systems, HAF008) was added to the membranes and incubated for 2 h at RT. Finally, membranes were washed with TBS-T and developed using SuperSignal West Pico Chemiluminescent Substrate (Life Technologies) or SuperSignal West Fempto Chemiluminescent Substrate (Life Technologies) and a LAS-3000 luminescent image analyzer (Fujifilm). The intensity of the bands was quantified using the FIJI software.

### 2.14 ELISA

For the quantification of IL-6 in cell culture supernatants, mouse IL-6 DuoSet ELISA (#DY406-ML, R&D Systems) was used according to manufacturer’s instructions.

### 2.15 Statistical analyses

The statistical analysis and data plotting were done with the GraphPad Prism 10 software. All values are expressed as mean ± SEM. Data were analyzed with Student’s t-test if normally distributed, Mann Whitney U-test if non-normally distributed, paired or non-paired depending on the experimental setup, or one-way analysis of variance (ANOVA) with *post hoc* Tukey’s test for multiple comparisons. p < 0.05 or adjp < 0.05 were set as significance levels.

### 2.16 Graphical presentation

Schemes were generated with Biorender.

## 3 Results

### 3.1 Inflammation induces itaconate production in microglia

**Figure 1:**
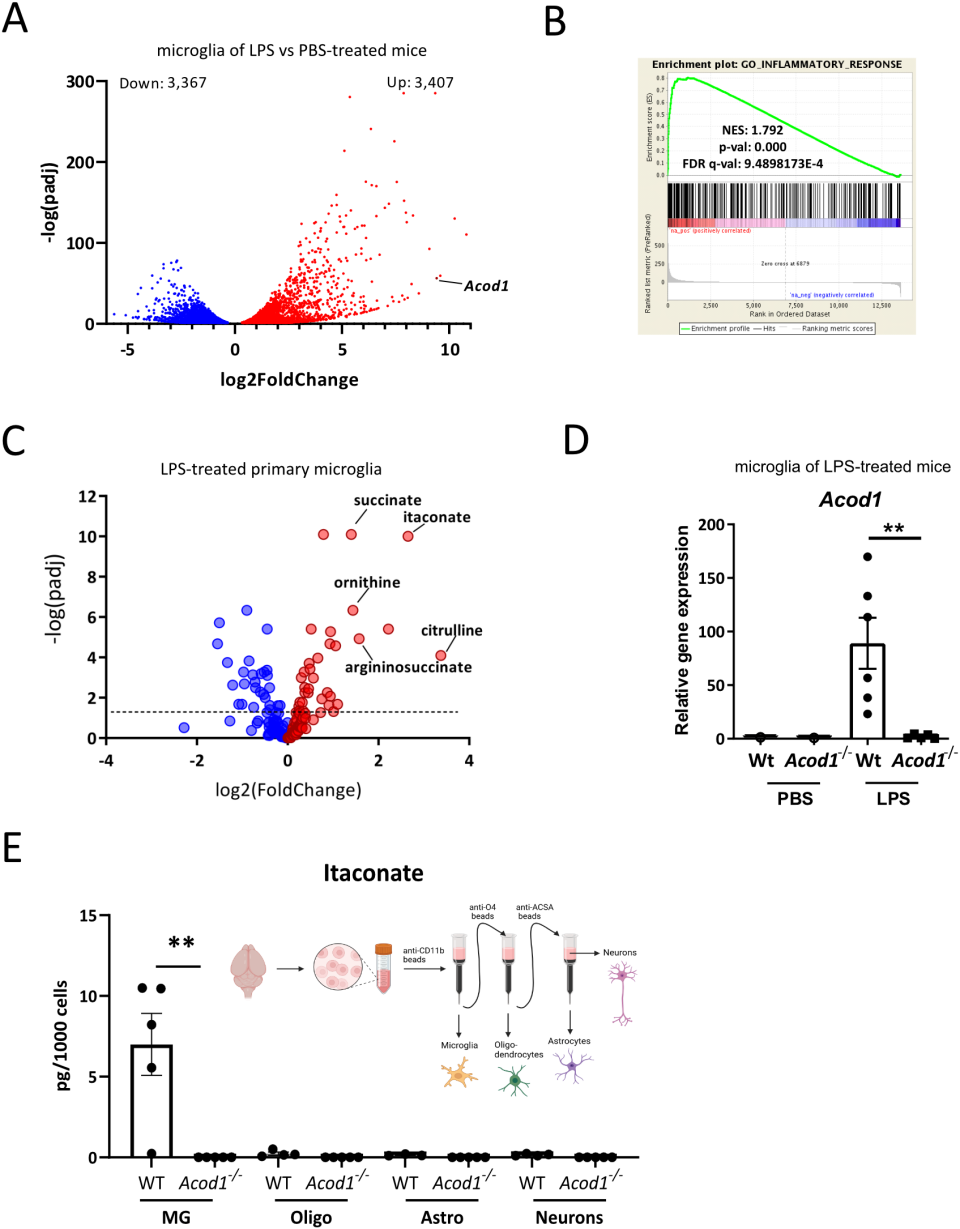
Inflammation induces itaconate production in microglia. **(A–C)** Bulk RNA-seq in sorted microglia (CD45^interm^CD11b^+^Ly6G^-^) from wt mice i.p. treated for 4 h with PBS or LPS (3 mg/kg)(n=3 mice per group). **(A)** Volcano plot showing differentially expressed genes. **(B)** Gene set enrichment analysis (GSEA) for inflammation-related genes. NES, normalized enrichment score; FDR, false discovery rate. **(C)** Volcano plot showing regulated metabolites in primary microglia treated or not for 24 h with LPS (100 ng/ml) (n=5 biological samples per group). **(D)** *Acod1* expression in microglia sorted from brains of wt and *Acod1^-/-^* mice treated for 16 h with LPS (3 mg/kg) or PBS (n=6 mice per group). **(E)** Itaconate amounts in isolated microglia (MG), oligodendrocytes (Oligo), astrocytes (Astro) and neurons from brains of wt and *Acod1*^-/-^ mice treated for 24 h with LPS (n=5 mice per group). **p < 0.01.

First, we validated that inflammation induces *Acod1* expression in microglia. To induce microglia inflammatory activation we treated wt C57BL/6J mice for 4 h i.p. with LPS, as previously described (27). Whole brain microglia were FACS sorted as CD45^interm^CD11b^+^Ly6G^-^ cells, distinguished from monocytes/macrophages (CD45^high^CD11b^+^Ly6G^-^) and neutrophils (CD45^high^CD11b^+^Ly6G^+^). Transcriptional changes in microglia from LPS- and PBS-treated mice were assessed by bulk RNA-seq (**Figure 1A**). In total, 3,407 genes were upregulated and 3,367 genes were downregulated in microglia of LPS-treated mice (**Figure 1A**) and, as expected, microglia of LPS-treated mice exhibited strong enrichment of inflammatory response-related gene sets, shown by gene set enrichment analysis (GSEA) (**Figure 1B**). *Acod1* was one of the top upregulated genes in microglia of LPS-treated mice (**Figure 1A**). These findings were confirmed *in vitro* in mouse primary microglia treated for 4 h with LPS by bulk RNA-seq analysis. LPS-treated primary microglia showed transcriptional reprograming and enrichment of inflammation-related gene sets, and *Acod1* was amongst the most upregulated genes in the LPS-treated cells (**Supplementary Figures 1A, B**). Upregulation of *Acod1* gene and protein expression was further verified by qPCR and western blot in primary microglia treated with LPS and IFN-γ (**Supplementary Figures 1C, D**).

Accordingly, itaconate and succinate were the most upregulated metabolites in primary microglia treated for 24 h with LPS, as shown by non-targeted metabolomics (**Figure 1C**). Succinate accumulation was in accordance with the inhibitory effect of itaconate on SDH and reduced expression of *Sdhb* and *Sdhd* in inflammatory microglia (RNAseq data, not shown), in agreement with previous reports (4, 5, 30, 39). Moreover, LPS treatment led to increased intracellular amounts of arginine metabolites, including ornithine, citrulline and argininosuccinate (**Figure 1C**). Increased citrulline and argininosuccinate levels indicate activation of the arginine biosynthesis pathway sustaining nitric oxide production in inflammatory macrophages (40, 41). Increased ornithine amounts are due to upregulation of arginase 1 and 2 (ARG1, ARG2) and facilitate polyamine and proline synthesis involved in resolution of inflammation and tissue recovery in the later stages of the inflammatory response (40).

Next, we asked which cell types produce itaconate in the brain upon inflammation. To this end, microglia and brain resident macrophages (CD11b^+^), oligodendrocytes (O4^+^), astrocytes (ACSA-2^+^) and neurons (negative for CD11b, O4 and ACSA-2) were sorted from wt and *Acod1^-/-^* mice treated for 24 h i.p. with LPS, and analyzed by LC-MS/MS (**Figures 1D, E**). Itaconate was detected in high amounts in CD11b^+^ cells (microglia/brain macrophages) but not in the other cell populations (oligodendrocytes, astrocytes, neurons), and its production in CD11b^+^ cells was completely blunted in ACOD1 deficient mice (**Figure 1E**).

Finally, we asked which inflammatory stimuli induce *Acod1* expression in microglia. To this end, BV2 microglia cells were treated for 4 h with different Toll like receptor (TLR) ligands, such as PAM3CSK4 (TLR1/TLR2 ligand), HKLM (TLR2 ligand), poly(I:C) (TLR3 ligand), FLA-ST (TLR5 ligand), FSL1 (TLR2/6 ligand), imiquimod (TLR7 ligand), ssRNA40/Lyovec (TLR8 ligand) and ODN2006 (TLR9 ligand), different cytokines, like M-CSF, GM-CSF, IL-1β, IL-6, TNF, IL-4, TGF-β, IL-10, IFN-γ, and LPS or LPS+IFN-γ, and *Acod1* expression was examined by qPCR. Out of the tested substances, LPS and LPS+IFN-γ most strongly induced *Acod1* expression. PAM3CSK4, HKLM, polyI:C, FLA-ST, FSL1, ODN2006, IL-1β, TNF and IFN-γ also upregulated *Acod1* expression (**Supplementary Figure 2**).

### 3.2 ACOD1 knockout enhances the inflammatory response of microglia

**Figure 2:**
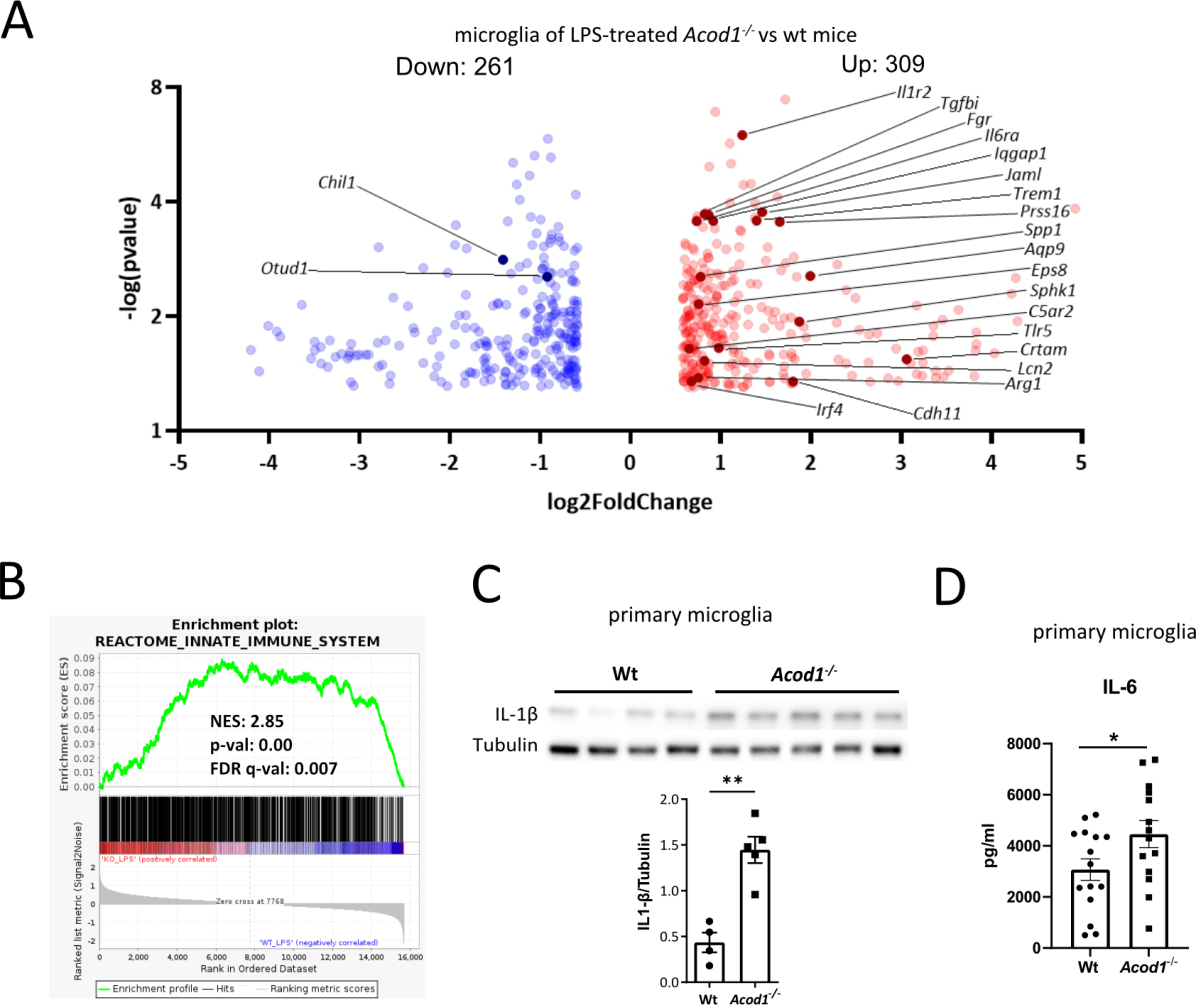
ACOD1 knockout enhances the inflammatory response of microglia. **(A, B)** Bulk RNA-seq in sorted microglia (CD45^interm^CD11b^+^Ly6G^-^) from *Acod1*^-/-^ and wt mice treated for 16 h with LPS (3 mg/kg) (n=3 mice per group). **(A)** Volcano plot showing differentially expressed genes. **(B)** GSEA for innate immune system-related genes. **(C)** Western blot analysis for IL-1β in *Acod1*^-/-^ and wt primary microglia treated for 24 h with LPS+IFN-γ and band intensity quantification using tubulin as a loading control (n=4-5). **(D)** IL-6 amounts in supernatants of *Acod1^-/-^* and wt microglia treated for 4 h with LPS (n=14-15). *p < 0.05, **p < 0.01.

Next, we examined the role of ACOD1 in microglia-mediated inflammation. To this end, *Acod1^-/-^* and littermate wt mice were treated i.p. with LPS, and 16 h later whole brain microglia were sorted as CD45^interm^CD11b^+^Ly6G^-^ cells and analyzed by bulk RNA-seq. In total, 309 genes were upregulated and 261 genes were downregulated in microglia of *Acod1^-/-^* compared to wt mice (**Figure 2A**). Upregulated genes included mediators of inflammation, such as interleukin 1 receptor type 2 (*Il1r2)*, interleukin 6 receptor subunit alpha (*Il6ra)*, secreted phosphoprotein 1 (*Spp1)*, *Arg1*, Triggering Receptor Expressed On Myeloid Cells 1 (*Trem1)*, Sphingosine Kinase 1(*Sphk1)*, Toll Like Receptor 5 (*Tlr5)*, Complement C5a Receptor 2 (*C5ar2)*, Cytotoxic And Regulatory T Cell Molecule (*Crtam)*, Interferon Regulatory Factor 4 (*Irf4)* and Cadherin 11 *(Cdh11)* (**Figure 2A**). Accordingly, GSEA analysis showed significant positive enrichment of gene sets related to the innate immune system in microglia of *Acod1^-/-^* compared to wt mice (**Figure 2B**). ACOD1 deficiency increased IL-1β and IL-6 levels in inflammatory microglia (**Figures 2C, D**), standing in accordance with its previously reported effects in LPS-treated macrophages (5). In contrast, ACOD1 deficiency did not increase LPS-induced *Tnf* expression (not shown), standing in agreement with other reports (5). Finally, validating the inhibitory effect of itaconate on SDH (5), ACOD1 deficiency reduced intracellular succinate amounts and the succinate/fumarate ratio in inflammatory primary microglia (**Supplementary Figures 3A, B**).

### 3.3 ACOD1 deficiency reprograms arginine metabolism

**Figure 3:**
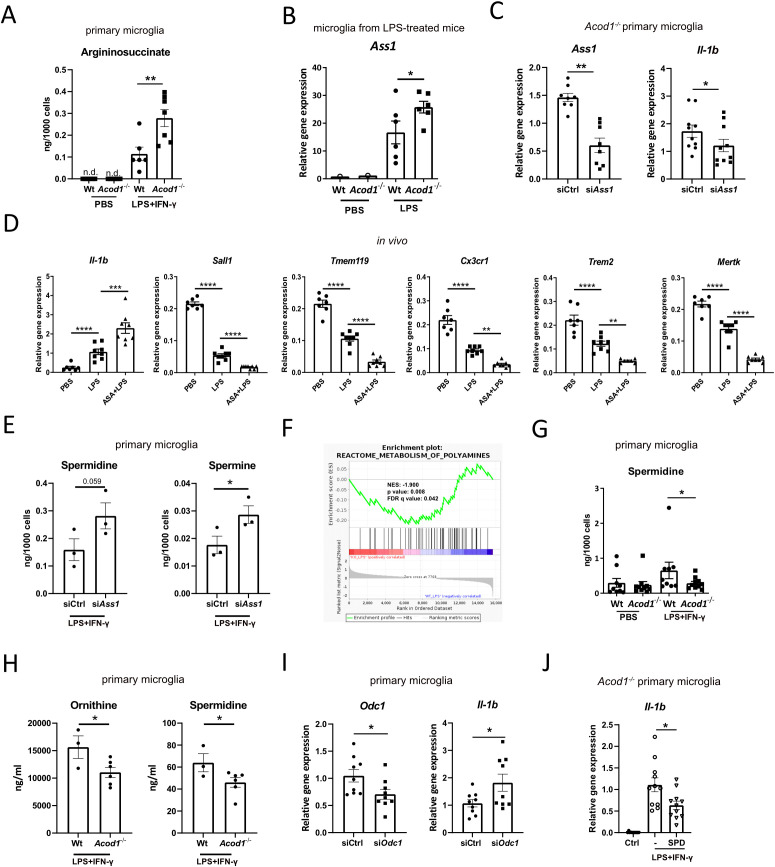
ACOD1 deficiency reprograms arginine metabolism. **(A)** Argininosuccinate amounts in primary *Acod1*^-/-^ and wt microglia treated for 24 h with LPS+IFN-γ or carrier (PBS) (n=6-7). **(B)** *Ass1* expression in microglia sorted from *Acod1*^-/-^ and wt mice treated for 16 h with PBS or LPS (3 mg/kg) (n=6 mice per group). **(C)** *Ass1* and *Il-1b* expression in primary *Acod1*^-/-^ microglia transfected for 24 h with si*Ass1* or siCtrl and treated with LPS+IFN-γ for 24 h (n=8-10). **(D)** mRNA expression of *Il-1b, Sall1, Tmem119, Cx3cr1, Trem2* and *Mertk* in microglia sorted from wt mice treated for 3 h with argininosuccinate (ASA) (50 mg/kg) and then for 4 h with LPS (1 mg/kg) (n=7–8 mice per group). **(E)** Spermidine and spermine amounts in wt primary microglia transfected with 30 nM si*Ass1* or siCtrl and treated with LPS+IFN-γ for 24 h (n=3). **(F)** GSEA for polyamine metabolism-related genes based on bulk RNAseq analysis in microglia sorted form LPS-treated *Acod1*^-/-^ and wt mice (n=3 mice per group). **(G)** Spermidine amounts in *Acod1*^-/-^ and wt primary microglia treated for 24 h with LPS+IFN-γ (n=9-10). **(H)** Ornithine and spermidine amounts in primary *Acod1*^-/-^ and wt microglia treated for 24 h with LPS+IFN-γ (n=3-6). **(I)** *Odc1* and *Il-1b* expression in primary wt microglia transfected for 48 h with 30 nM si*Odc1* or siCtrl and then treated for 4 h with LPS+IFN-γ (n=9). **(J)** *Il-1b* expression in primary *Acod1*^-/-^ microglia treated for 24 h with spermidine (SPD), LPS+IFN-γ or respective controls (n=11). *p < 0.05, **p < 0.01, ***p<0.001, ****p<0.0001. n.d.: non-detectable.

Arginine metabolism is a key metabolic hub in the regulation of macrophage immune responses (40). Proinflammatory macrophages metabolize arginine to nitric oxide and citrulline, and in turn, citrulline can be used to regenerate arginine in order to sustain nitric oxide production (40, 41). This requires the conversion of citrulline to argininosuccinate by argininosuccinate synthase 1 (ASS1) and the break-down of argininosuccinate to arginine and fumarate by the argininosuccinate lyase (ASL) (40). The citrulline-nitric oxide cycle is activated in inflammatory macrophages and ASS1-mediated intracellular citrulline depletion is required for the proinflammatory response of macrophages (41, 42). We showed that citrulline and argininosuccinate accumulate in LPS-stimulated microglia (**Figure 1D**). Interestingly, ACOD1 deficiency enhanced argininosuccinate amounts in inflammatory microglia (**Figure 3A**). Accordingly, microglia sorted from brains of LPS-treated *Acod1^-/-^* mice displayed increased *Ass1* expression compared to microglia of wt mice (**Figure 3B**). In accordance with the fact that argininosuccinate synthesis is linked to inflammation (40, 41), siRNA silencing of *Ass1* reduced *Il-1b* expression in *Acod1*^-/-^ primary microglia (**Figure 3C**). In order to validate the proinflammatory role of argininosuccinate *in vivo*, we treated wt mice i.p. with argininosuccinate (50 mg/kg) prior to LPS treatment and whole brain microglia were sorted 4 h after the LPS injection. Argininosuccinate treatment further increased *Il-1b* expression and enhanced the LPS-mediated suppression of the homeostatic genes *Sall1*, *Tmem119* and *Cx3cr1*, and phagocytic genes *Trem2* and Mer tyrosine kinase (*Mertk*) in microglia (**Figure 3D**) (43, 44). These data suggest that ACOD1 deficiency enhances argininosuccinate production, which promotes IL-1β-mediated inflammation and reduces homeostatic microglia features.

In inflammatory macrophages iNOS and ARG1 compete to convert arginine to citrulline and ornithine, respectively (40). While citrulline is the substrate for ASS1, ornithine is metabolized by ornithine decarboxylase 1 (ODC1) to putrescine; the latter is the rate-limiting reaction for the synthesis of polyamines, spermidine and spermine (40). Polyamines suppress inflammation and facilitate resolution of inflammation (40, 45, 46). In order to validate the antagonism between ASS1 and polyamine biosynthesis, we transfected wt primary microglia with *Ass1* siRNA under LPS+IFN-γ stimulation, and measured spermidine and spermine in the cell lysates by LC-MS/MS. Indeed, *Ass1* siRNA silencing increased spermidine and spermine amounts in inflammatory microglia (**Figure 3E**). Moreover, in accordance with increased argininosuccinate production, gene expression related to polyamine metabolism was downregulated in microglia of *Acod1^-/-^* LPS-treated mice (**Figure 3F**). Accordingly, intracellular and secreted amounts of spermidine and ornithine were decreased in *Acod1^-/-^* compared to wt primary microglia under LPS+IFN-γ treatment (**Figures 3G, H**). In order to validate the anti-inflammatory role of polyamine biosynthesis, we transfected wt primary microglia with *Odc1* siRNA under LPS+IFN-γ stimulation and analyzed *Il-1b* expression by qPCR. Indeed, *Odc1* siRNA silencing (despite its low deletion efficiency) increased *Il-1b* expression in inflammatory microglia (**Figure 3I**). Finally, in accordance with its previously reported anti-inflammatory effects, spermidine reduced *Il-1b* expression in inflammatory *Acod1^-/-^* microglia (**Figure 3J**) (40, 45). Collectively, these data demonstrate that in inflammatory microglia ACOD1 deficiency tilts arginine metabolism to argininosuccinate synthesis, which promotes inflammation to the expense of polyamine biosynthesis that mitigates inflammation.

### 3.4 ACOD1 regulates arginine metabolism via ACLY

**Figure 4:**
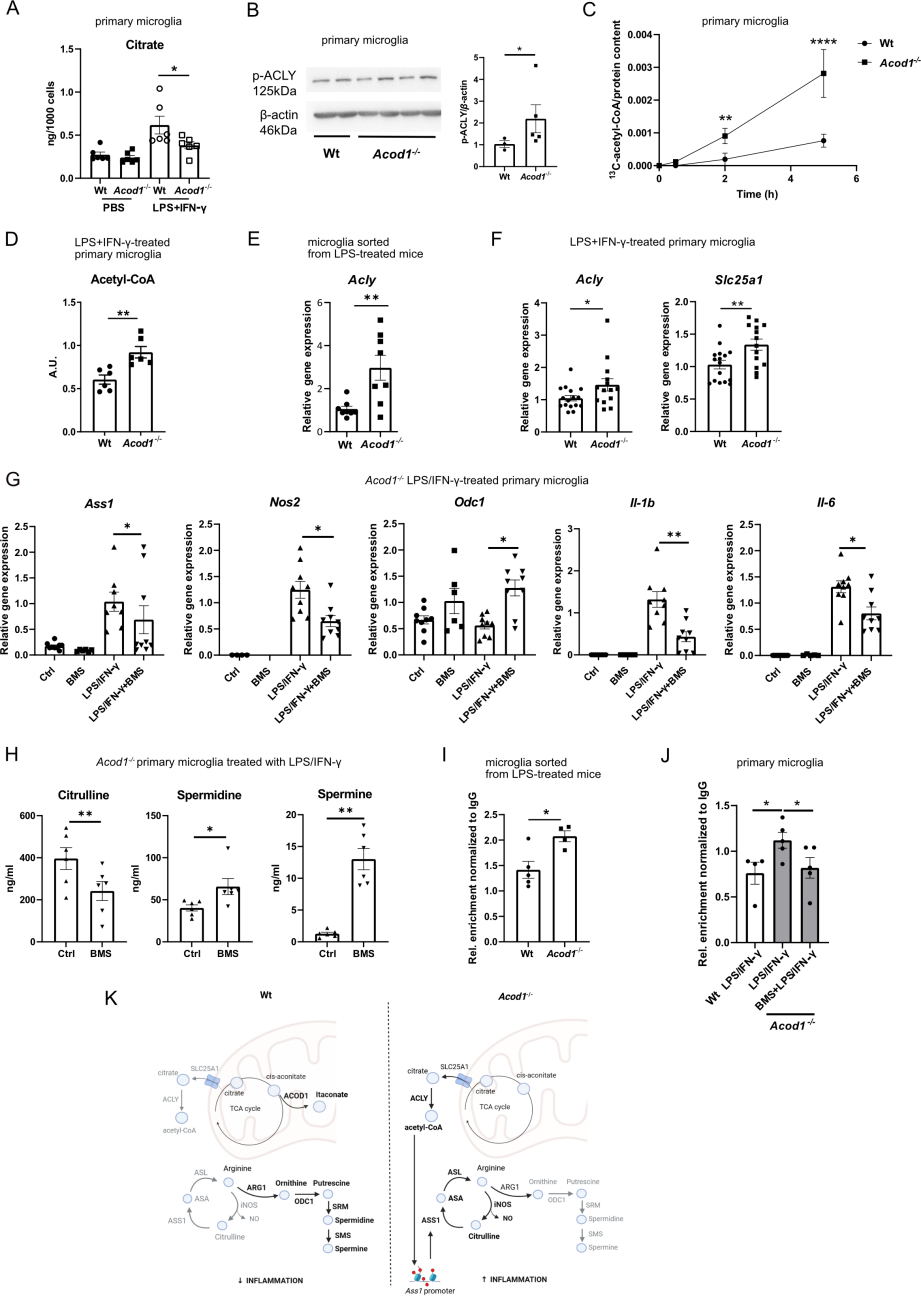
ACOD1 regulates arginine metabolism via ACLY. **(A)** Citrate amounts in *Acod1*^-/-^ and wt microglia treated for 24 h with PBS or LPS+IFN-γ (n=6). **(B)** Western blot for phosphorylated ACLY in *Acod1*^-/-^ and wt microglia treated for 24 h with LPS+IFN-γ using β-actin as a loading control and band intensity quantification (n=3-5, 2 experiments). **(C)** *Acod1*^-/-^ and wt primary microglia were incubated with ^13^C-citrate for the indicated times, ^13^C-acetyl-CoA was measured in the cell lysates by LC–MS/MS and normalized to the protein content (n=3-5). **(D)** Acetyl-CoA levels in *Acod1*^-/-^ and wt primary microglia treated for 24 h with LPS+IFN-γ (n=6). **(E)** *Acly* expression in microglia sorted from *Acod1*^-/-^ and wt mice treated for 16 h with LPS (3 mg/kg) (n=8 mice per group). **(F)** *Acly* and *Slc25a1* mRNA expression in *Acod1*^-/-^ and wt microglia treated for 24 h with LPS+IFN-γ (n=14-16). **(G)** *Ass1*, *Nos2*, *Odc1*, *Il-1b* and *Il-6* expression in *Acod1^-/-^* microglia treated for 24 h with BMS303141 (20 μM), LPS+IFN-γ or respective controls (n=8-14). **(H)** Citrulline, spermidine and spermine amounts in *Acod1^-/-^* microglia treated for 24 h with LPS+IFN-γ and BMS303141 (20 μM) or carrier (n=6). **(I)** Abundance of H3K9ac marks in the *Ass1* gene promoter in microglia sorted from LPS-treated *Acod1*^-/-^ and wt mice, shown by Cut&Tag (n=4-5). **(J)** Abundance of H3K9ac marks in the *Ass1* gene promoter in *Acod1*^-/-^ and wt primary microglia treated for 24 h with LPS+IFN-γ and BMS303141 (20 μM) or carrier, shown by Cut&Tag (n=4-5). **(K)** Schematic presentation of the hypothesis. *p < 0.05, **p < 0.01, ****p<0.0001.

ACOD1 deficiency reduced citrate levels in inflammatory microglia (**Figure 4A**). Citrate is transported via SLC25A1 from the mitochondria to the cytoplasm, where it is converted by ACLY to acetyl-CoA (47). ACOD1 may regulate availability of citrate by consumption of its downstream metabolite cis-aconitate. Therefore, we asked whether ACOD1 regulates ACLY activity in inflammatory microglia. Indeed, ACOD1 deficiency increased ACLY phosphorylation (**Figure 4B**) and enhanced the conversion of ^13^C-citrate to ^13^C-acetyl-CoA, indicating enhanced ACLY activity (**Figure 4C**). Consequently, acetyl-CoA was elevated in *Acod1*^-/-^ compared to wt inflammatory microglia (**Figure 4D**). In accordance, microglia sorted from LPS-treated *Acod1*^-/-^ mice displayed increased *Acly* expression compared to microglia from wt counterparts (**Figure 4E**). Similarly, *Acly* and *Slc25a1* expression was higher in *Acod1*^-/-^ compared to wt primary microglia treated with LPS+IFN-γ (**Figure 4F**).

Next, we asked whether elevated ACLY activity is linked to arginine metabolism reprograming in ACOD1 deficient inflammatory microglia. To this end, *Acod1*^-/-^ LPS+IFN-γ-treated microglia were treated with the specific ACLY inhibitor BMS303141 and the expression of genes playing a key role in arginine metabolism (*Ass1*, *Nos2*, *Odc1*) was examined. BMS303141 decreased *Ass1* and *Nos2* expression and increased *Odc1* expression suggesting that ACLY promotes the ASS1/iNOS-driven proinflammatory arm of arginine metabolism, while downregulating polyamine biosynthesis (**Figure 4G**). Accordingly, BMS303141 decreased citrulline and increased spermidine and spermine amounts in *Acod1*^-/-^ LPS+IFN-γ-treated primary microglia (**Figure 4H**). Consequently, BMS303141 abolished *Il-1b* and *Il-6* expression in *Acod1*^-/-^ LPS+IFN-γ-treated microglia (**Figure 4G**).

Finally, we set out to explore the mechanism through which ACLY regulates arginine metabolism. In inflammatory macrophages, acetyl-CoA synthesized by ACLY is used as a substrate for histone acetylation in proinflammatory genes, thereby promoting their expression (48). We asked whether ACLY may promote activating histone acetylation marks, such as H3K9ac, in the *Ass1* promoter in ACOD1 deficient microglia. Cut&Tag analysis showed increased abundance of H3K9ac in the *Ass1* gene promoter in microglia sorted from ACOD1 deficient mice treated with LPS (**Figure 4I**). Moreover, BMS303141 reduced the amounts of H3K9ac in the *Ass1* promoter in *Acod1*^-/-^ LPS+IFN-γ-treated primary microglia (**Figure 4J**).

Altogether, these data suggest that ACOD1 deficiency promotes ACLY activity, which upregulates *Ass1* expression by increasing histone acetylation in the *Ass1* gene promoter. Consequently, this promotes argininosuccinate/citrulline/nitric oxide over polyamine synthesis fostering inflammation (**Figure 4K**) (48–50).

## 4 Discussion

The ACOD1-itaconate axis has emerged as a significant regulator of inflammation, as demonstrated by numerous studies in macrophages (2, 5, 6). However, less is known about its role in microglia, the resident macrophage-like cells of the brain. We confirmed that similarly to macrophages, microglia upregulate ACOD1 expression in response to inflammation, which provides negative feedback on the inflammatory response, standing in accordance with other reports (23–26). LPS was identified as the most potent inducer of ACOD1 expression compared to different TLR ligands and cytokines. Moreover, *Acod1* and itaconate were selectively upregulated by LPS in microglia among glial and neuronal cells in the mouse brain.

Some recent studies have addressed the role of ACOD1 in microglia. Traumatic brain injury (TBI) in mice triggered *Acod1* expression in microglia (51). Microglia-specific ACOD1 deficiency exacerbated TBI-associated inflammation, neurodegeneration and neurological dysfunction and distorted microglial bioenergetics, while the itaconate analogue 4-octyl itaconate (4-OI) restored microglial oxidative metabolism (51). In a model of intracerebral hemorrhagic stroke, microglia-specific ACOD1 deficiency reduced erythrocyte clearance thereby aggravating disease, while itaconate or 4-OI restored microglial phagocytic capacity (52). In injury-induced brain ischemia ACOD1 deficient mice displayed aggravated neuroinflammation, blood-brain barrier disruption and brain injury (53). In spinal cord injury, *Acod1* expression was elevated in spinal cords, while overexpression of *Acod1* or treatment with itaconate led to suppression of LPS-induced inflammation in microglia (26). 4-OI and dimethyl itaconate (DMI) were suggested to halt experimental autoimmune encephalomyelitis (EAE) progression in mice (54, 55), although the ameliorating effect of 4-OI in EAE was disputed by others (56). Moreover, *Acod1^-/-^* deficient mice presented greater microglia density and allograft inflammatory factor 1 (AIF1) reactivity in the CA1 region and dentate gyrus upon systemic LPS stimulation (23). Accordingly, systemic DMI treatment restrained microgliosis in the hippocampus of *Toxoplasma gondii*-infected mice (57).

Here, we focused on the immunometabolic role of ACOD1 in microglia. We demonstrate that ACOD1 deficiency enhanced ACLY activity, potentially due to increased mitochondrial citrate availability, upregulated the expression of the *Slc25a1* mitochondrial citrate transporter and consequently increased citrate-derived acetyl-CoA production. Increased ACLY activity in LPS-treated macrophages was previously shown to promote *Il-6* and *Il-1b* expression via upregulation of histone acetylation in their promoter regions (48, 58). Moreover, itaconate-bearing lipid nanoparticles targeting atherosclerotic plaques reduced H3K27ac marks in inflammatory genes in myeloid cells (59). In accordance with these reports, we found that ACOD1 deficiency increased the abundance of H3K9ac marks in the *Ass1* gene promoter and upregulated *Ass1* expression and argininosuccinate amounts, while these effects were downregulated by ACLY inhibition. An alternative mechanism leading to increased argininosuccinate production in *Acod1^-/-^* microglia could be mediated by the lifting of SDH inhibition due to itaconate depletion, promoting the metabolic pathway fumarate – malate – aspartate – argininosuccinate, which fuels the aspartate-argininosuccinate shunt (60). Hence, increased substrate availability combined with upregulated ASS1 expression in *Acod1^-/-^* microglia may foster the aspartate-argininosuccinate shunt, thereby sustaining arginine regeneration and nitric oxide production (40, 41, 60). The ASS1-argininosuccinate axis promoted *Il-1b* expression, standing in accordance with previous studies showing that ASS1-mediated depletion of citrulline, which inhibits JAK2-STAT1 signaling, is required for host defense against bacterial infection (42). Accordingly, previous reports showed that iNOS inhibits inflammasome activation and promotes inflammasome tolerance in synergy with itaconate in LPS-treated macrophages (61). Hence, ASS1 and argininosuccinate arise as key factors in promoting the inflammatory response, especially in the context of ACOD1 deficiency, in microglia and potentially other tissue resident macrophage(-like) cells. Finally, our data suggest that tilting arginine metabolism towards argininosuccinate production controls polyamine biosynthesis. Polyamines mediate anti-inflammatory and pro-resolving effects (45, 46, 62, 63); hence, impediment of polyamine synthesis may additionally favor the proinflammatory phenotype of ACOD1 deficient microglia.

Taken together, these findings demonstrate a so far overlooked immunometabolic connection, in which the ACOD1/itaconate axis regulates ACLY activity and maintains a balanced arginine metabolism, thereby orchestrating the inflammatory responses of microglia. This is a concept that may also apply to other tissue resident macrophages.

## Data Availability

The datasets presented in this study can be found in online repositories. The names of the repository/repositories and accession number(s) can be found below: MSV000099684 (MassIVE, https://doi.org/doi:10.25345/C5K06XD70) and GSE299665 (GEO).
