# Supplementary material for: ACOD1 regulates microglial arginine metabolism and inflammatory responses
Source: Front Immunol. 2026 Mar 16;17:1731962. doi: 10.3389/fimmu.2026.1731962 (PMC13033549; doi:10.3389/fimmu.2026.1731962)
Supplement: Supplementary file 1 [file DataSheet1.pdf]

Supplementary data

Supplementary Figure 1. LPS induces *Acod1* expression in primary microglia. (A-C) RNA-seq in primary microglia treated for 4 h with LPS or carrier (PBS) (n=4). **(A)** Volcano plot showing differentially expressed genes. **(B)** GSEA for inflammation-related genes. **(C)** *Acod1* mRNA expression in primary microglia cells treated for 4 h with LPS+IFN-γ or carrier (PBS) (n=6). **(D)** Western blot analysis for ACOD1 in primary microglia cells treated for 24 h with LPS+IFN-γ or control solution (PBS), using Vinculin as a loading control. **p < 0.01.

Supplementary Figure 2. Effect of different TLR ligands and cytokines on *Acod1* expression in microglia. *Acod1* mRNA expression in BV2 cells treated or not for 4 h with various TLR ligands (n=6) **(A)** or cytokines (n=7) **(B)**. *p < 0.05, **p < 0.01, ***p < 0.001, ****p < 0.0001.

Supplementary Figure 3. Succinate and succinate/fumarate in primary *Acod1*$^{-/-}$ and wt microglia treated for 24 h with LPS+IFN-γ or carrier (PBS) (n=6), **p < 0.01.
