# Supplementary figures and images for: ACOD1 regulates microglial arginine metabolism and inflammatory responses
Source: Front Immunol. 2026 Mar 16;17:1731962. doi: 10.3389/fimmu.2026.1731962 (PMC13033549; doi:10.3389/fimmu.2026.1731962)

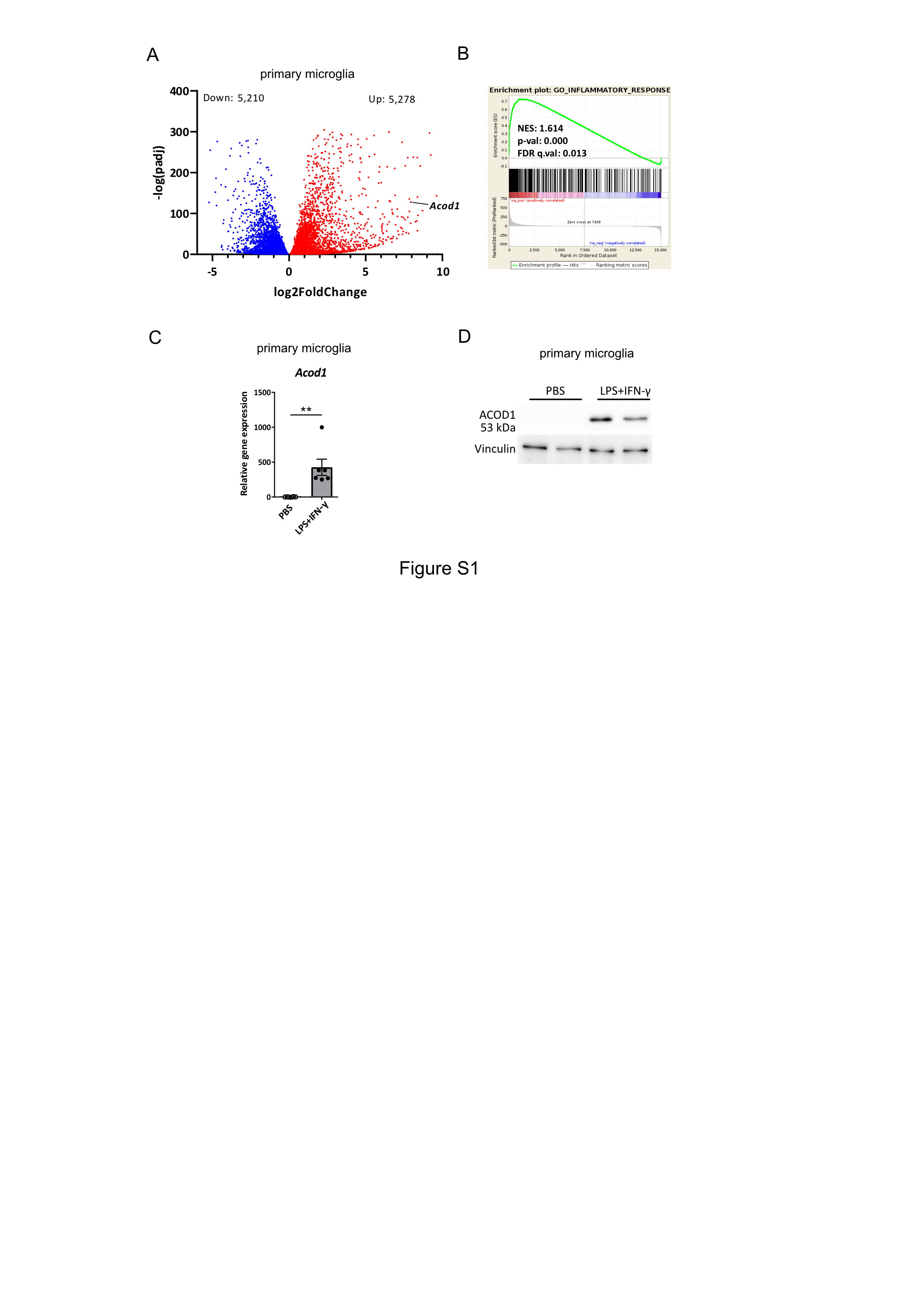

Supplement: Supplementary file 3 [file Image1.tiff]

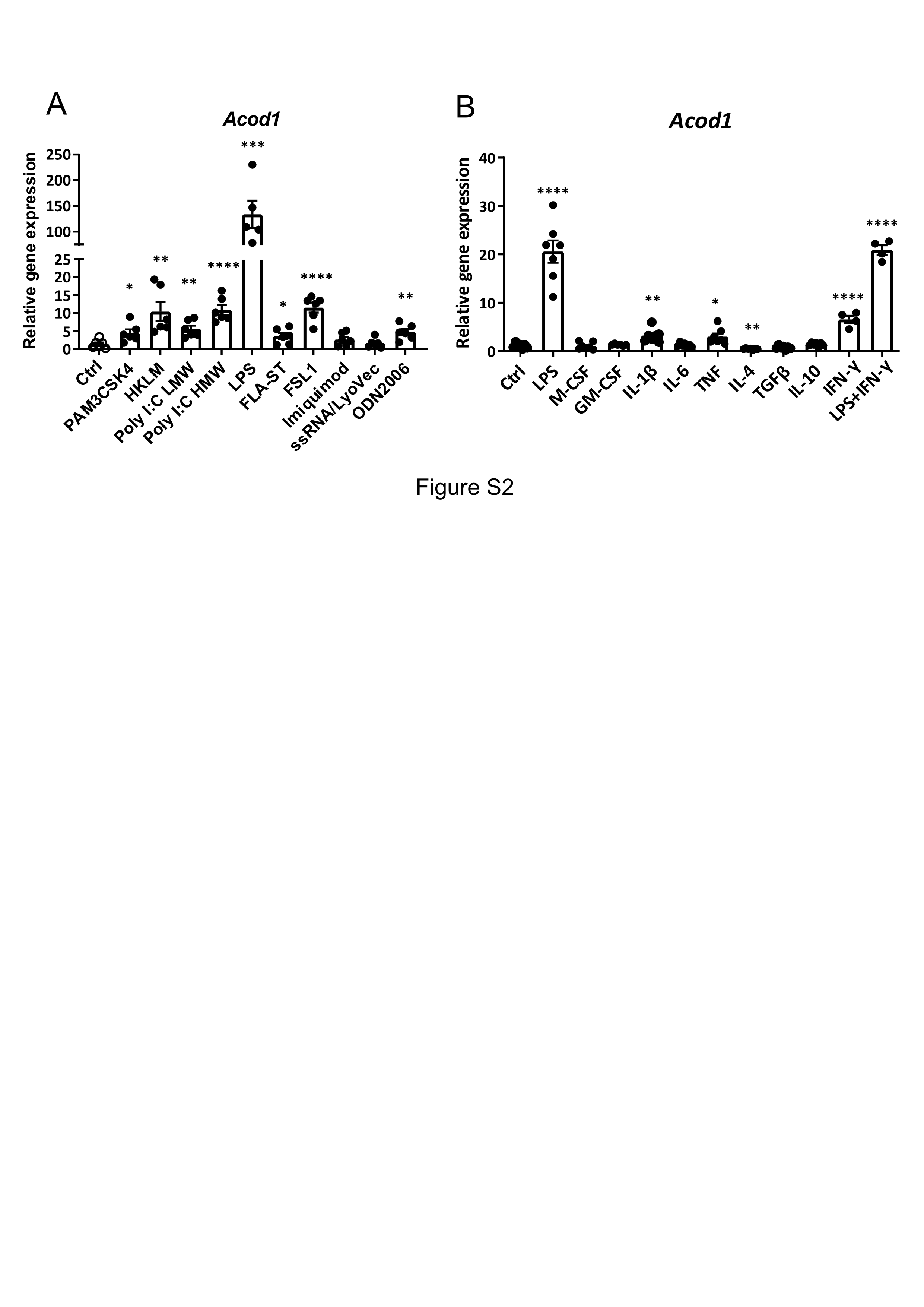

Supplement: Supplementary file 4 [file Image2.tiff]

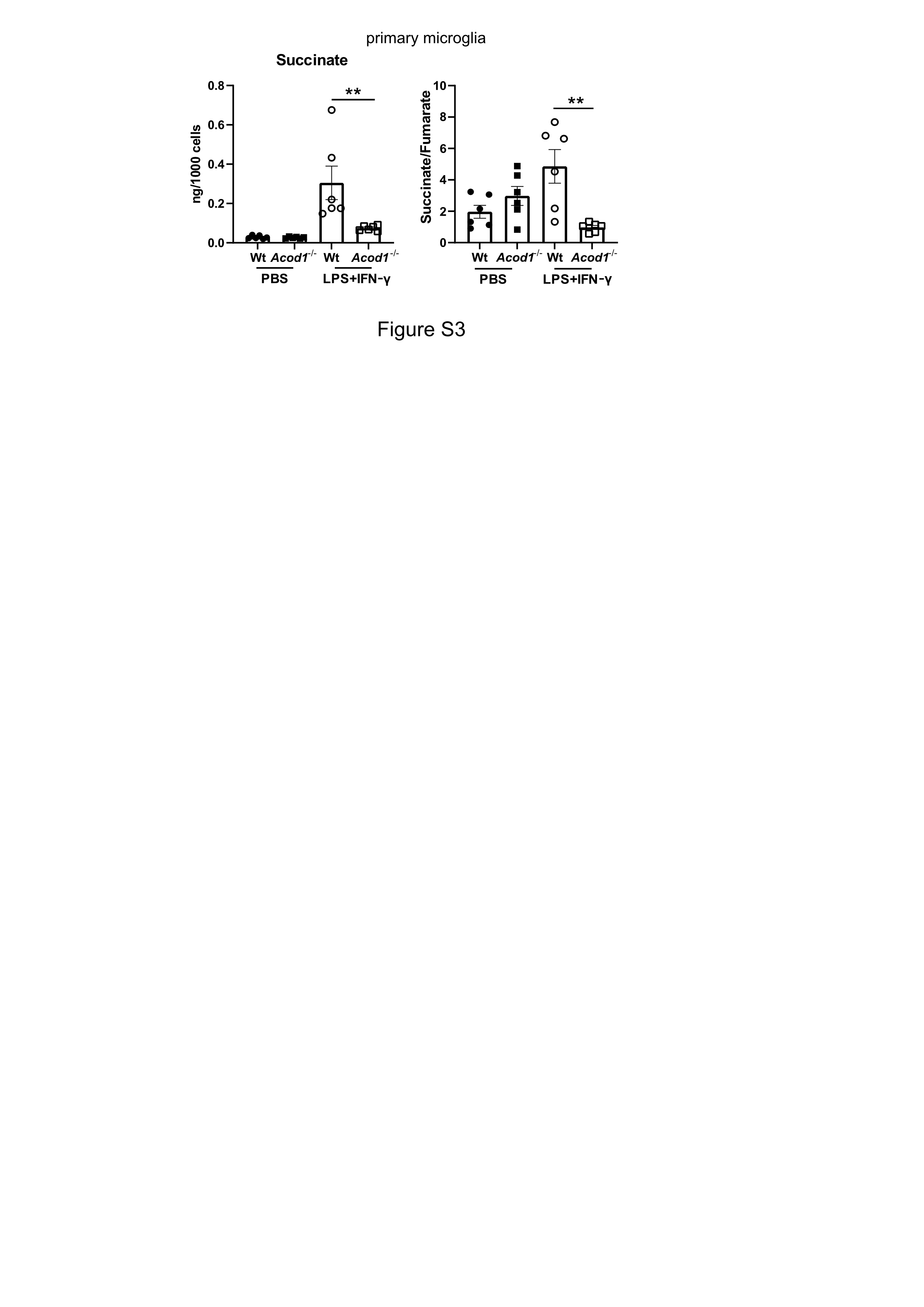

Supplement: Supplementary file 5 [file Image3.tiff]
